# Role of pulmonary intravascular macrophages in endotoxin-induced lung inflammation and mortality in a rat model

**DOI:** 10.1186/1465-9921-9-69

**Published:** 2008-10-24

**Authors:** Sukhjit S Gill, Sarabjeet S Suri, Kyathanahalli S Janardhan, Sarah Caldwell, Tanya Duke, Baljit Singh

**Affiliations:** 1 Department of Veterinary Biomedical Sciences, University of Saskatchewan, Saskatoon, SK S7N5B4, Canada; 2 Department of Small Animal Clinical Sciences, University of Saskatchewan, Saskatoon, SK S7N5B4, Canada

## Abstract

**Background:**

Bile-duct ligated (BDL) rats recruit pulmonary intravascular macrophages (PIMs) and are highly susceptible to endotoxin-induced mortality. The mechanisms of this enhanced susceptibility and mortality in BDL rats, which are used as a model of hepato-pulmonary syndrome, remain unknown. We tested a hypothesis that recruited PIMs promote endotoxin-induced mortality in a rat model.

**Methods:**

Rats were subjected to BDL to induce PIM recruitment followed by treatment with gadolinium chloride (GC) to deplete PIMs. Normal and BDL rats were treated intravenously with *E. coli *lipopolysaccharide (LPS) with or without GC pre-treatment followed by collection and analyses of lungs for histopathology, electron microscopy and cytokine quantification.

**Results:**

BDL rats recruited PIMs without any change in the expression of IL-1β, TNF-α and IL-10. GC caused reduction in PIMs at 48 hours post-treatment (P < 0.05). BDL rats treated intravenously with *E. coli *LPS died within 3 hours of the challenge while the normal LPS-treated rats were euthanized at 6 hours after the LPS treatment. GC treatment of rats 6 hours or 48 hours before LPS challenge resulted in 80% (1/5) and 100% (0/5) survival, respectively, at 6 hours post-LPS treatment. Lungs from BDL+LPS rats showed large areas of perivascular hemorrhages compared to those pre-treated with GC. Concentrations of IL-1β, TNF-α and IL-10 were increased in lungs of BDL+LPS rats compared to BDL rats treated with GC 48 hours but not 6 hours before LPS (P < 0.05).

**Conclusion:**

We conclude that PIMs increase susceptibility for LPS-induced lung injury and mortality in this model, which is blocked by a reduction in their numbers or their inactivation.

## Background

Bile duct ligated (BDL) rats show biliary cirrhosis and are used as a model to study hepato-pulmonary syndrome which occurs in 10–15% of human patients with cirrhosis and portal hypertension, has no treatment and causes significant mortality [1,2]. BDL rats have increased vascular translocation of Gram negative bacteria, increased blood levels of TNF-α, endothelin-1 and endotoxins as well as recruitment of PIMs [3,4]. PIMs have been linked to an increase in TNF-α expression and iNOS activity in BDL rats [2,3]. Although a relationship between PIMs and sensitivity of BDL rats to endotoxin-induced mortality has been speculated [5], a direct link between the two is yet to emerge.

PIMs are unique inflammatory cells, which are normally present in sheep, cattle, goat and horse but not in humans, dogs, rats and mice [6,7]. The species without PIMs, compared to those with PIMs, tolerate large dosages of endotoxin without showing significant pulmonary vascular responses, inflammation and edema [8-11]. The PIMs are credited with removal of majority of blood-borne endotoxins and bacteria even following injection in hepatic portal vein [12]. We and others have shown that removal of PIMs with gadolinium chloride or clodronate inhibits endotoxin-induced lung inflammation[13,14]. Interestingly, PIM recruitment is observed in species normally devoid of these cells under experimental physiological stresses such as liver injury induced by chronic BDL and intraperitoneal infection with *E. coli *[5,15,16]. Although the biology of recruited PIMs is poorly understood, PIM recruitment may increase host susceptibility for lung inflammation [15].

The role of recruited PIMs in endotoxin-induced inflammation in BDL rats, which are used as a model for hepatopulmonary syndrome, is largely obscure. Therefore, we investigated the biology of PIMs in BDL rats with an aim to determine if PIM depletion protects against endotoxin-induced mortality and lung inflammation. The experimental data show that BDL induces recruitment of PIMs and their depletion or inactivation protects BDL rats from *E. coli *LPS induced lung inflammation and mortality.

## Materials and methods

### Animals

The experimental protocols were approved by the University Protocol Review Committee on Animal Care and Supply, and experiments were conducted according to the Canadian Council on Animal Care Guidelines. Specific pathogen free 350–400 gram male Sprague-Dawley rats were procured from Charles River Laboratories, Canada. Rats were acclimatized for a period of one week before the experiment.

### Experiment 1

BDL was performed on rats as previously described [1,5]. Briefly, rats were anesthetized by intraperitoneal administration of xylazine (20 mg/kg) and ketamine (100 mg/kg). A mid line incision was made, the common bile duct was identified and ligated at two points 5 mm apart and resected in between the two ligatures (N = 10). In sham operated animals (N = 4), the common bile duct was identified and separated from the surrounding soft tissue without ligation and resection. After recovery from anesthesia, rats had free access to water and rat chow. After 4 weeks, five of the BDL rats were treated with gadolinium chloride (GC;10 mg/kg tail vein) and remaining five were used as BDL controls. The rats were euthanized 48 hours after the GC treatment under xylazine-ketamine anesthesia and cardiac exsanguation. Five rats were euthanized without any surgery or treatment.

### Experiment 2

Control and BDL (4 weeks after the surgery) rats (n = 6/group) were administered *E. coli *lipopolysaccharide (LPS) intravenously (0.1 mg/kg iv). In another group, 10 BDL rats were administered GC (10 mg/kg i.v) and were challenged with *E. coli *LPS either 6 hours (N = 5) or 48 hours (n = 5) after GC treatment. All animals were euthanized 6 hours after the LPS treatment.

### Lung tissue collection and processing

Lungs were collected as previously described [15]. The right bronchus was ligated followed by right lung removal and freezing in liquid nitrogen. The left lung was instilled with 2% paraformaldehyde with 0.1% glutaraldehyde under 20 cm water pressure and ligated at the level of the trachea to prevent backflow. The ligated left lung was removed and fixed in 4% paraformaldehyde for 24 hours. Following fixation, the lung was cut into several pieces of 3 mm thickness. From each lung, pieces numbered 2, 4 and 6 were collected for light microscopy. Tissues collected for light microscopy were processed and embedded in paraffin. Sections (five micron) were cut onto the poly-L-Lysine coated slides and used for hematoxylin and eosin staining and immunostaining.

Pieces of the left lung were fixed in 2% paraformaldehyde containing 0.1% glutaraldehyde for 3 hours at 4°C and processed for embedding in LR White followed by polymerization under ultraviolet light for immuno-electron microscopy. One micron sections were prepared and stained with toluidine blue to select appropriate areas for preparation of thin (100 nm) sections, which were placed on nickel grids.

### Immunohistochemistry for monocytes/macrophages

Immunohistochemical methods have been described previously [15]. Briefly, lung sections from all the rats in each of the groups were deparaffinized and rehydrated followed by quenching of endogenous peroxidase. Sections were treated with pepsin (2 mg/ml 0.01 N hydrochloric acid) to unmask antigens, to 1% bovine serum albumin to block non-specific sites and to primary anti-macrophage ED-1 antibody (1:100) for 60 minutes followed by appropriate horseradish peroxidase-conjugated secondary antibody (1:100) for 30 minutes. The color was developed with a commercial kit (Vector Laboratories, Canada). The staining controls included incubation of sections with only secondary antibody, isotype matched antibodies or with only color development reagents. Another control was to stain some of the sections with anti-von Willebrand Factor (vWF) antibody (1:200), which recognizes vascular endothelium. Finally, sections were counter-stained with methyl green.

### Quantification of ED-1 positive cells

The methods have been described previously [15]. An observer, who was blinded to the identity of treatment groups, counted the ED-1 positive cells in alveolar septa. The observer obtained counts of septal cells stained with ED-1 antibody from 10 fields at X100 in lung sections from all the rats used in the experiments.

### Immuno-gold electronmicroscopy

Immuno-gold electron microscopy methods have been described elsewhere [15]. Briefly, the sections were blocked with 1% bovine serum albumin and 0.1% Tween-20 in tris-buffered saline; pH 7.9 for 30 minutes followed by incubation with primary (ED-1, TNF-α, IL-1β and IL-10) antibodies for one hour and appropriate gold conjugated secondary antibodies for one hour. The sections were stained with 2% aqueous uranyal acetate and then lead citrate.

### Reverse transcriptase-polymerase chain reaction

Total RNA was extracted from lungs of three rats from each group tissues of rat by sequential extraction with TRIzol reagent (Invitrogen, ON, Canada) followed by treatment with RNase-free DNase (Qiagen, ON, Canada) and purification on RNeasy mini columns (Qiagen) according to the manufacturer's instructions. Integrity of RNA was confirmed by agarose gel electrophoresis and RNA was quantified by spectrophotometric analysis. Superscript III one-step RT-PCR system with Platinum *Taq *DNA polymerase (Invitrogen) was used to detect expression of IL-1β: 5'-TTGCCCGTGGAGCTTC-3' 5'-CGGGTTCCATGGTG AAC-3'), TNF-α: 5'-GCACAGAAAGCATGATCC-3' 5'-GTGGGTGAGGAGCACAT-3') and IL-10 (5'-GCTGCGACGCTGTCAT-3' 5'-GCGCTGAG CTGTTGCT-3') in lung tissues from various treatments. Reactions were performed as directed by the manufacturer. Each reaction was performed using 10 ng of total RNA and thermocycler was programmed for reverse-transcription at 55°C for 30 min, initial denaturation of the cDNA at 94°C for 2 min, 30 amplification cycles, each of which consisted of 94°C for 15 sec, 59°C for 30 sec, and 68°C for 1 min followed by a final extension at 68°C for 5 min. To ensure lack of DNA contamination 2 units of Platinum Taq DNA polymerase was substituted for the Superscript III RT/Platinum *Taq *mixture in the reaction. A negative control reaction consisted of all the components of the reaction mixture except RNA. Amplified PCR products were electrophoresed on a 1 % TAE-agarose gel, stained with ethidium bromide and photographed under UV light.

### Myeloperoxidase assay

Myeloperoxidase (MPO) assay was performed on lung tissues from three rats from each group as described previously [17]. Briefly, Lung tissues were homogenized in 50 mM Hepes (pH 8.0) containing 0.5% CTAC and cell-free extract was stored at -20°C till further use. Samples were diluted in phosphate citrate buffer (pH 5.0). To the 75 μl sample, equal volume of the substrate (977.5 μl/mL of TMB, 20.0 μl/mL of 6 mM Resorcinol and 2.5 μl/mL of 3% H_2_O_2_) was added and after 2 minutes, the reaction was stopped by adding 150 μl of stop solution (1 M H_2_SO_4_). For zero minutes, 75 μl of sample was added to 150 μl of cold stop solution containing 75 μl of substrate. Microplate was read at 450 nm and the change in OD/min was calculated.

### Enzyme-linked immunosorbent assay

For enzyme-linked immunosorbent assay (ELISA), frozen lung tissues from three rats from each group were homogenized in HBSS solution containing protease inhibitor cocktail and centrifuged at 4°C for 20 minutes at 15000 g. Supernatants were collected and stored at -80°C till further use. Microtitre plates were coated with 50 μl of capture antibody diluted with sodium phosphate buffer and incubated at 4°C overnight. Next day, following wash and incubation with blocking buffer (1% BSA in PBS) for one hour, wells were washed with PBST (PBS containing 0.05%Tween-20). Standards and samples (100:l; diluted in PBST containing 1% BSA) were added to the wells and plate was incubated at 37°C for 2 hours. After removing standards and samples, wells were washed with PBST and detection antibody (100:l; diluted in PBS containing 1% BSA) was added. Plates were incubated at 37°C for one hour. After washing wells with PBST, streptavidin-HRP (100:l; 1:2500 in PBS containing 1% BSA) was added to each well and incubated at room temperature for 30 minutes. Wells were washed thoroughly with PBST, TMB substrate (100:l) was added and incubated in dark at room temperature for 20 minutes. On color development, reaction was stopped by adding 50:l of 0.5 M sulfuric acid. Microplates were read at 450 nm.

### Statistical analysis

Analysis was done using a statistical software package (SPSS 12.0 for windows). Differences among groups were compared using one-way analysis of variance followed post-hoc group comparisons. Mortality data were compared with Fischer's test. Statistical significance was accepted at p < 0.05.

## Results

### Effect of BDL

#### Response to BDL

Rats became dull and inactive within two days of ligation of their bile ducts. Their food and water intake decreased and all rats lost up to 50–100 grams of weight over one week after the surgery. Although rats started eating and drinking normally and regained their body weights by 4 weeks after BDL, their urine and mucous membranes became progressively icteric. Nearly four weeks post-BDL, a swelling was evident upon palpation in the anterior abdomen of most of the rats as a result of proximal dilatation of ligated bile duct. Sham operated rats continued to grow normally even after surgery.

#### PIM recruitment

**Figure 1:**
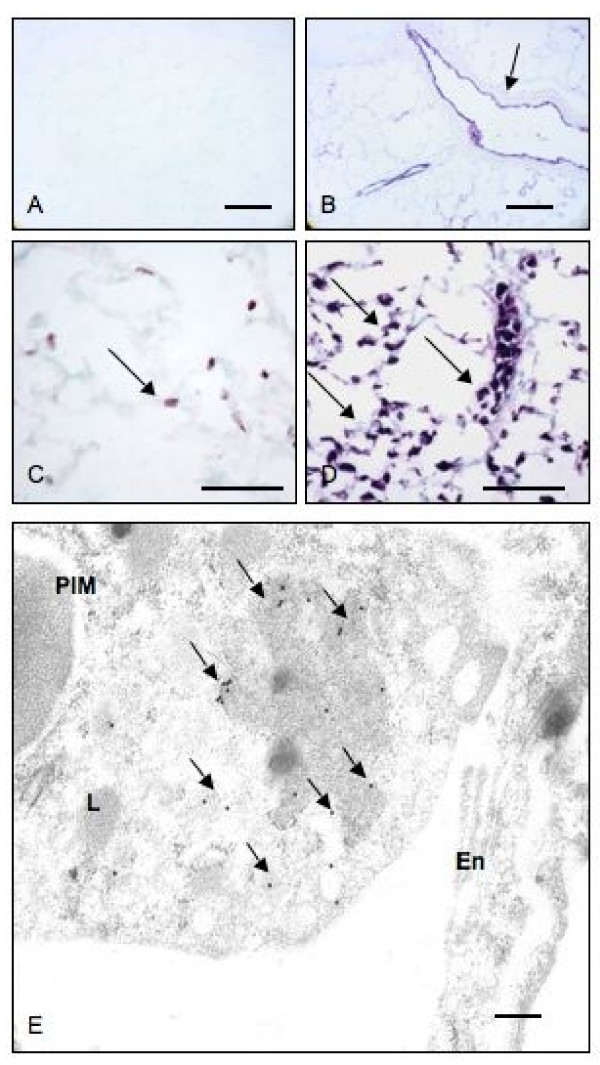
**PIM recruitment: Lung section stained with only secondary antibody (A) show no reaction while anti-vWF antibody stained vascular endothelium (arrow).** Lung sections from control rats (C) contained occasional septal ED1-positive cells (arrow) while those from BDL rats had numerous reactive cells in the septa (arrows). Immuno-electron micrograph (E) shows gold labeling (arrows) in the cytoplasm as an indication of ED-1 staining in a PIM. En: Endthelium; PIM: pulmonary intravascualr macrophage; L: lysosome. Bar: A-B: 50 μm; C-D: 100 μm; E: 1 μm.

**Figure 2:**
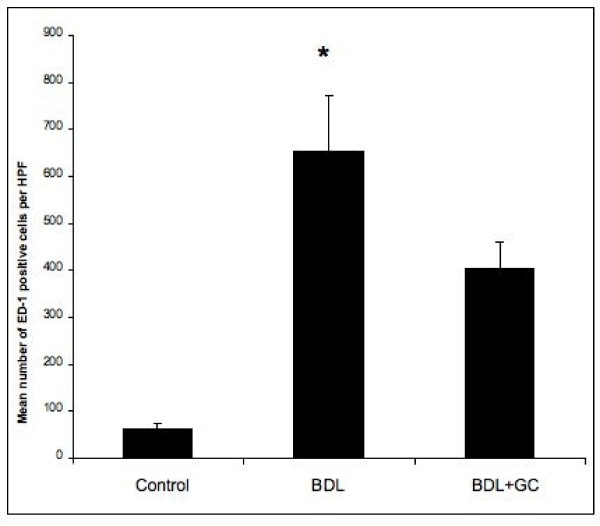
Numerical counts of PIMs: Septal ED-1 positive cells showed increase in PIMs in BDL rats compared to the normal rats or at 48 hours after GC treatment of BDL rats (*: P < 0.05).

Histologic examination showed normal lung morphology in control rats (no surgery) and sham-operated rats and increased numbers of mononuclear cells in the alveolar septa (data not shown). Immunohistochemistry with ED-1 antibody, which recognizes rat monocytes/macrophages, stained numerous septal monocytes/macrophages in lungs of BDL rats compared to the normal rats (Figures 1A–D). Immuno-electron microscopy confirmed the intravascular location, adherence to endothelium and ED-1 reactivity of septal macrophages (Figure 1E). Once the intravascular location of ED-1 positive cells was determined with immuno-electron microscopy, we counted these cells in lung sections under a light microscope. BDL rats showed more ED-1 positive cells in their alveolar septa compared to control rats (*p = 0.002*). GC treatment of BDL rats depleted ED-1 positive cells (*p = 0.05*) (Figure 2).

#### Expression of IL-1β, TNF-α and IL-10

We detected mRNA for IL-1β, TNF-α and IL-10 in lung homogenates from rats from control, BDL and BDL+GC group. There were no differences in protein concentrations of these three cytokines in lungs of rats from three groups (data not shown).

### Recruited PIMs, lung inflammation and mortality

#### Response to LPS challenge

Sham-operated rats did not show signs of stress following LPS treatment and were euthanised at 6 hours post-LPS treatment (Table 1). In contrast, all of the BDL rats (N = 6) upon challenge with LPS became dull and inactive, showed labored breathing, piloerection, defecation and urination before dying within 3 hours of the treatment. Interestingly, all the BDL rats (N = 5) that were treated with GC 48 hours before the LPS challenge survived till 6 hours after the LPS treatment. Furthermore, 4 out of the 5 BDL rats treated with GC 6 hours prior to the LPS challenge survived up to 6 hours post-LPS treatment. The mortality in BDL+LPS group was significantly higher (P < 0.05) compared to all other groups while there were no differences between BDL rats treated with GC before the LPS challenge, the control rats and control rats given only LPS (P > 0.05).

**Table 1:** Mortality in rats (* = P < 0.05 compared to all other groups).

**Experimental group**	**Number of animals**	**Mortality within 6 hrs of LPS challenge**	
		
		**Number of rats**	**%**
LPS	N = 5	0	0
BDL+LPS	N = 6	6*	100
BDL+GC(6H)+LPS	N = 5	1	20
BDL+GC(48H)+LPS	N = 5	0	0

#### Histopathology, neutrophil and PIM recruitment

**Figure 3:**
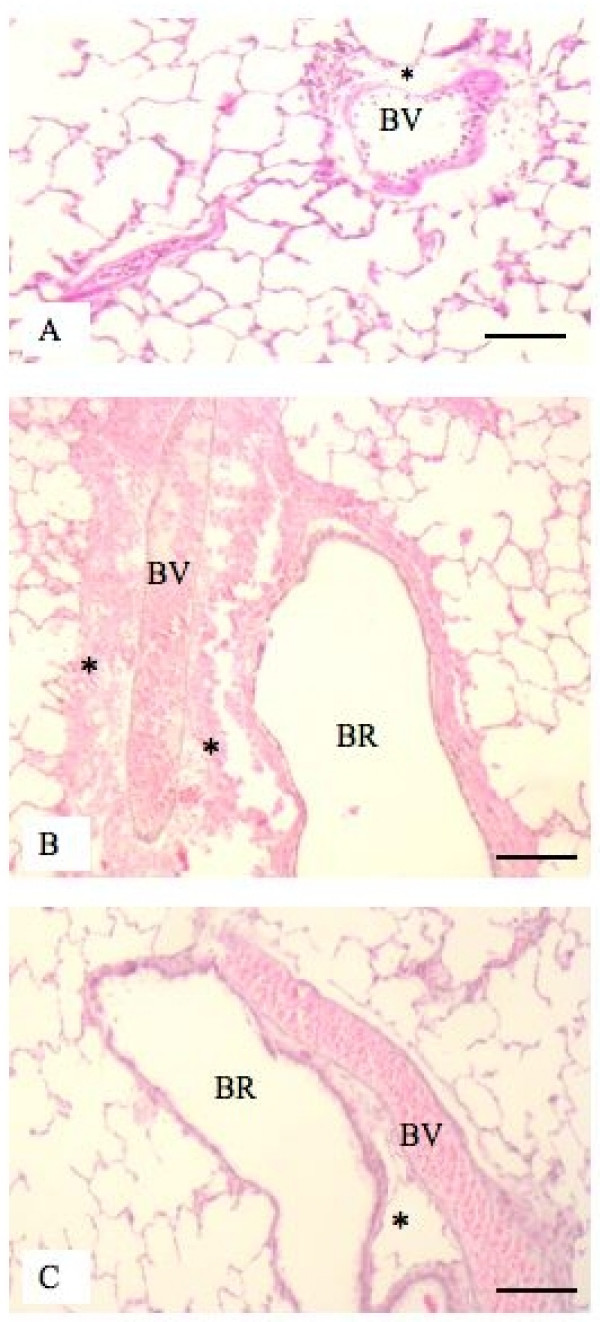
Histopathology of lungs: H&E staining showed normal histology of lungs from control rats (A) but hemorrhages in perivascular space (*) in lungs of BDL rats treated with LPS (B). Perivascular hemorrhage was absent in lungs of control (A) and BDL rats treated with GC before LPS challenge (C). BR: Bronchiole; BV: Blood vessel. Bar: 100 μm.

**Figure 4:**
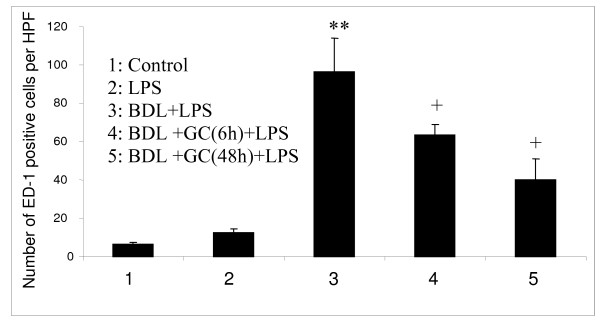
PIM quantification: BDL rats treated with LPS showed more numbers of PIMs (**) compared to all other groups while both GC-treated BDL groups had higher number of PIMs (+) compared to the control and the LPS groups (P < 0.05).

We observed perivascular hemorrhages in BDL rats treated with LPS compared to those BDL rats treated with GC prior to LPS treatment (Figure 3). Interestingly, MPO assay showed no differences in neutrophil recruitment in lungs among various treatment groups (data not shown). We found significantly more ED-1 positive cells in BDL+LPS rats compared to all other groups including those BDL rats treated with GC before LPS challenge (P < 0.05; Figure 4).

#### Expression of IL-1β, TNF-α and IL-10

**Figure 5:**
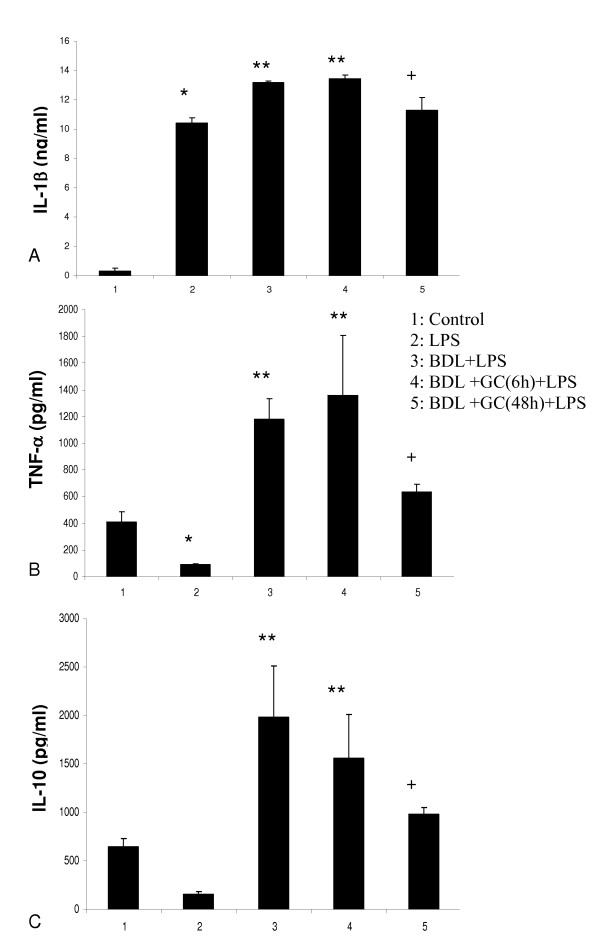
**Cytokine expression: IL-1β (A) TNF-α (B) and IL-10 (C) protein concentrations in lung homogenates of various groups.** BDL rats treated with LPS showed higher concentrations (**) of all the measured cytokines compared to all other groups. + indicates more concentrations than the control and the LPS group while * means more than the control rats. P < 0.05.

**Figure 6:**
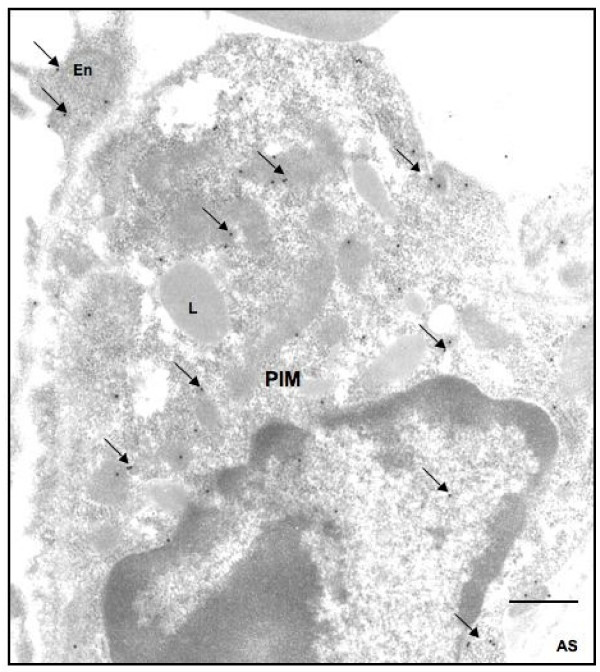
**Ultrastructural localization of TNF-α: Immuno-gold electron microscopy showed TNF-α labeling (arrows) in cytoplasm of a pulmonary intravascular macrophage (PIM) and endothelium (En).** L: Lysomse; AS: Alveolar space. Bar: 1 μm.

BDL rats challenged with LPS had significantly higher concentrations (P < 0.05) of IL-1β (Figure 5A), TNF-α (Figure 5B) and IL-10 (Figure 5C) in their lungs compared with the control rats, normal rats given only LPS and BDL rats treated with GC 48 hours but not 6 hours before the LPS treatment. Control rats and normal rats treated with LPS had similar lung concentrations of IL-10 (Figure 5C). Immuno-gold electron microscopy showed labeling of PIMs for TNF-α (Figure 6), IL-1β and IL-10 (data not shown).

## Discussion

There is relatively little data on the biology of PIMs compared to other lung macrophages. We have used BDL rats, which recruit PIMs and have been used by others as a model of hepato-pulmonary syndrome, to investigate the role of PIMs in endotoxin-induced mortality and lung inflammation. The data show that PIM depletion as well as inactivation prevents mortality and lung inflammation in LPS-treated BDL rats.

### GC reduces the numbers of recruited PIMs in BDL rats

BDL rats have been used to study the biology of PIMs as well as the mechanisms of hepato-pulmonary syndrome in human patients with liver cirrhosis [4,5,18,19]. Similar to previous studies, we also found recruitment of ED-1 positive PIMs in BDL rats. ED-1 antibody binds to a single chain 110 kD glycoprotein expressed in lysosomes of monocytes/macrophages. Due to spatial resolution limits of the light microscope, we used electron microscopy to confirm that ED-1 reactive cells were in septal capillaries, attached to the endothelium and contained lysosomes. Therefore, these cells fulfill ultrastructural and molecular phenotypic criteria for PIMs [6,20]. Recruited PIMs in BDL rats form tighter adhesion with capillary endothelium compared to the transiently recruited PIMs following intraperitoneal *E. coli *infection [15,16,21]. We observed that GC significantly reduced PIM numbers in BDL rats without affecting expression of IL-1β, TNF-α and IL-10. GC quickly inactivates macrophages before inducing apoptosis in them [22,23] and has been used to remove PIMs *in vivo *in sheep, cattle and horses and alveolar macrophages in mice and rat, [11,14,24-26]. Interestingly, histologic evaluation showed lack of reduction in macrophages in liver of GC-treated animals. This relative lack of impact of GC on liver cells including macrophages could be due to sequestration of GC, similar to other vascular substances such as endotoxins as shown previously, in PIMs and little availability for liver macrophages in PIM-containing species [12]. Taken together, these light and electron microscopic data confirm identity of PIMs, and a reduction in their numbers with GC treatment in BDL rats provides us a model to compare host response in BDL rats in the absence or presence of PIMs.

### PIM reduction protects against LPS-induced mortality in BDL rats

We delineated role of PIMs by comparing LPS-induced lung inflammation and mortality in BDL rats and GC-treated BDL rats. The dramatic finding was 100% and 80% survival in BDL rats that were treated with GC 48 hours and 6 hours, respectively, prior to the LPS challenge compared to 100% mortality in LPS-treated BDL rats. In addition to the reduced mortality, there was a reduction in the severity of histological signs of inflammation including recruitment of ED-1 positive cells in the BDL rats treated with GC before the LPS treatment. Although mortality in LPS-treated BDL rats was similar to that previously reported [5], the striking impact of reduction in PIM numbers at 48 hours post-GC treatment on the mortality in BDL rats is novel. The reduced mortality observed in BDL rats treated with GC 6 hours before the LPS challenge is intriguing. In vitro data show that GC rapidly inactivates macrophages before inducing apoptosis in them by 24–48 hours [22,23]. Although it is difficult to determine inactivating effects of GC on PIMs in vivo, we speculate that improved survival in the 6 hour group may be due to inactivation of PIMs by the GC. Taken together, the evidence that PIM depletion or inactivation protects against LPS-induced mortality shows PIMs' central role in mortality in this model.

We used the LPS treatment because there is evidence of translocation of endotoxins from the gut of BDL rats, which have been used as a model for hepato-pulmonary syndrome by other investigators [3,4]. Endotoxin-induced inflammation and shock is characterized by a "cytokine storm" [27]. Macrophages interact with endotoxins via Toll-like receptor 4 and are one of the major sources of proinflammatory cytokines in endotoxic shock [28-30]. Therefore, we believe that transient reduction of a major cellular source of multiple cytokines such as a PIM instead of neutralization of a single cytokine in a disease is more beneficial and more rational. The role of recruited PIMs is indirectly highlighted by the recent linkage between susceptibility to diseases such as hepatopulmonary syndrome, for which BDL rats used as a model, and the MCP-1 gene [31]. We observed increased expression of MCP-1 protein in lungs of BDL rats (unpublished data). Even though PIM reduction/inactivation had beneficial effect in LPS-treated BDL rats and in itself is mechanistic, we further examined the lung concentrations of IL-1β, TNF-α and IL-10. These three inflammatory cytokines play critical roles in endotoxin-induced pathophysiology [32-38] First, we observed ultrastructural localization of IL-1β, TNF-α and IL-10 in PIMs in LPS-treated BDL rats. Second, we found a significant reduction in the concentrations of these important and highly relevant cytokines in PIM-depleted LPS-treated BDL rats compared to LPS-treated BDL rats, which could be a reason for 100% survival in these rats. Although mortality was also significantly attenuated in the 6 hour pre-treatment group, there was no reduction in the concentrations of the measured cytokines in lung homogenates. The reasons for this discrepancy between cytokine levels in the lung and reduced mortality are not readily apparent. Based on the GC's previously demonstrated ability to inactivate macrophages, we speculate that GC-induced PIM inactivation may have prevented secretion of these cytokines and resulted in intracellular retention leading to their measurements with ELISA on lung homogenates. Another reason for reduced mortality in GC-treated BDL rats challenged with LPS may be attenuation of massive perivascular hemorrhages observed in BDL rats challenged with LPS. An intriguing observation was lack of effect of GC treatment on neutrophil recruitment indicated by similar MPO levels in lung homogenates of various groups. Although neutrophils have been linked to increased inflammation, tissue damage and mortality, the cytokines such as IL-1β can directly induce shock and mortality [32,39]. We do believe that additional experiments focusing on the role of individual cytokine such as IL-1β and TNF-α through their blockade or the use of specific gene knockout mice will shed more light on the mechanisms of this inflammatory process. Taken together, these data show that PIM depletion leading to reduced concentrations or intracellular retention of IL-1β, TNF-α and IL-10 results in the beneficial effects.

These new data demonstrate a critical role of recruited PIMs in endotoxin-induced mortality in a BDL rats. It is striking that depletion of a single inflammatory cell results in remarkable reduction in key inflammatory cytokines, inhibition of lung inflammation and mortality. Although significant effort is invested in therapeutic targeting of single cytokines, these data show the attraction of therapeutic targeting of a cell such as PIMs.

## Competing interests

The authors declare that they have no competing interests.

## Authors' contributions

SSG conducted the experiments, analysed the data and participated in the preparation of the first draft of the manuscript, SSS performed RTPCR and helped with ELISA, KSJ assisted in surgeries and MPO assay, SC helped with immuno-electron microscopy, TD is helped in supervision of the project and read the manuscript and BS is the principal investigator who participated in the study design, data analyses and manuscript preparation.
